# Tick-borne encephalitis in a naturally infected sheep

**DOI:** 10.1186/s12917-017-1192-3

**Published:** 2017-08-22

**Authors:** Brigitte Böhm, Benjamin Schade, Benjamin Bauer, Bernd Hoffmann, Donata Hoffmann, Ute Ziegler, Martin Beer, Christine Klaus, Herbert Weissenböck, Jens Böttcher

**Affiliations:** 1 Bavarian Animal Health Service, Senator-Gerauer-Straße 23, 85586 Poing, Germany; 2 Institute of Diagnostic Virology, Friedrich-Loeffler-Institute, Südufer 10, 17493 Greifswald-Insel Riems, Germany; 3 Institute of Novel and Emerging Infectious Diseases, Friedrich-Loeffler-Institute, Südufer 10, 17493 Greifswald-Insel Riems, Germany; 4 Institute of bacterial Zoonoses and Infections, Friedrich-Loeffler-Institute, Naumburger Straße 96 a, 07743 Jena, Germany; 5 Institute of Pathology and Forensic Veterinary Medicine, University of Veterinary Medicine, Veterinärplatz 1, 1210 Vienna, Austria

**Keywords:** Tick-borne encephalitis virus, TBEV, Histopathology, RT-qPCR, ELISA, *Ixodes ricinus*

## Abstract

**Background:**

Tick-borne encephalitis (TBE) is the most important viral tick borne zoonosis in Europe. In Germany, about 250 human cases are registered annually, with the highest incidence reported in the last years coming from the federal states Bavaria and Baden-Wuerttemberg. In veterinary medicine, only sporadic cases in wild and domestic animals have been reported; however, a high number of wild and domestic animals have tested positive for the tick-borne encephalitis virus (TBEV) antibody.

**Case presentation:**

In May 2015, a five-month-old lamb from a farm with 15 Merino Land sheep and offspring in Nersingen/Bavaria, a TBEV risk area, showed impaired general health with pyrexia and acute neurological signs. The sheep suffered from ataxia, torticollis, tremor, nystagmus, salivation and finally somnolence with inappetence and recumbency. After euthanasia, pathological, histopathological, immunohistochemical, bacteriological, parasitological and virological analyses were performed. Additionally, blood samples from the remaining, healthy sheep in the herd were taken for detection of TBEV antibody titres. At necropsy and accompanying parasitology, the sheep showed a moderate to severe infection with Trichostrongylids, Moniezia and Eimeria species. Histopathology revealed mild to moderate necrotising, lymphohistiocytic and granulocytic meningoencephalitis with gliosis and neuronophagia. Immunohistochemistry for TBEV was negative. RNA of a TBEV strain, closely related to the Kumlinge A52 strain, was detected in the brain by quantitative reverse transcriptase polymerase chain reaction (RT-qPCR) and subsequent PCR product sequencing. A phylogenetic analysis revealed a close relationship to the TBEV of central Europe. TBEV was cultured from brain tissue. Serologically, one of blood samples from the other sheep in the herd was positive for TBEV in an enzyme-linked immunosorbent assay (ELISA) and in a serum neutralisation test (SNT), and one was borderline in an ELISA.

**Conclusion:**

To the authors’ knowledge this is the first report of a natural TBEV infection in a sheep in Europe with clinical manifestation, which describes the clinical presentation and the histopathology of TBEV infection.

## Background

Tick-borne encephalitis (TBE), caused by tick-borne encephalitis virus (TBEV), a *Flavivirus*, has been recognised for decades in Europe and some parts of Asia as an important viral zoonosis with between 5352 (2008) and up to 12,733 (1996) human cases annually [1]. In almost all cases, TBEV is transmitted by two types of ticks: *Ixodes (I.) ricinus,* which are found mainly in Western Europe and in Germany, and *I. persulcatus,* which are found in Eastern Europe and Siberia. However, an alimentary infection via raw milk or raw milk products from ruminants in the viraemic phase may also occur rarely [2, 3]. In Germany, TBE is a notifiable human disease with an average of 250 cases per year reported annually [4, 5]. TBEV circulates between vector ticks and competent hosts in so-called natural foci in a patchy distribution, whose geographic extension as a rule is strictly limited and can be very small [6]. In Germany, TBE risk areas are defined by the incidence of autochthonous human cases as the official statistical tool and are published every year [4, 5]. The TBEV infection risk in humans seems to be influenced by a combination of landscape and climatic variables as well as host-species dynamics [7]. Nearly 90% of all human cases occurred in the federal states of Bavaria and Baden-Wuerttemberg.

In veterinary medicine, clinical cases of TBE with neurological symptoms are rarely described, but have been reported in dogs for more than 30 years [8–10], as well as in horses [11–13] and monkey [14, 15]. In ruminants, like cows, goats and sheep as well as wild species such as roe deer and red foxes, TBEV antibody titres are observed without clinical symptoms [3, 16–19]. Very rarely, single clinical cases were registered, for example, in a goat in Switzerland [20] and a mouflon (*Ovis ammon musimon*) in Austria [21].

In this report, we describe the clinical manifestation of TBEV infection in a sheep with severe neurological symptoms, raised in a pasture in a district in Bavaria (GMS N 48°; O 10°; 456 m) defined as a TBE risk area [5]. Potential differential etiologies were ruled out (*Listeria monocytogenes*, *Borna disease virus*, *West Nile virus*, *Louping ill virus*).

## Case presentation

In May 2015, a five-month-old lamb from a farm with 15 Merino Land sheep showed impaired general health with pyrexia (41.2 °C) and acute neurological signs. The sheep suffered from ataxia, torticollis, tremor, nystagmus, salivation and finally somnolence with inappetence and recumbency. The veterinarian suspected listeriosis. The remaining sheep were healthy. The animal was euthanised and submitted for pathological examination. Samples of the brain stem, cerebellum, medulla oblongata, pons, cerebral cortex, hippocampus and thalamus were fixed in 10% buffered formalin for 48 h and subsequently embedded in paraffin wax. Sections of 4 μm thickness were mounted on glass slides and stained with haematoxylin and eosin (HE) and examined histologically.

A swab was taken from the brain stem and cultured for the suspected infection with *Listeria monocytogenes.* Twenty-four hours of aerobic incubation at 37 °C on Columbia Agar supplemented with 5% sheep’s blood as well as enrichment on Listeria enrichment broth and Fraser enrichment broth with subsequent use of Oxford Listeria agar and Brilliance™ Listeria agar (Oxoid, Wesel, Germany) showed no evidence of a Listeria infection.

Immunohistochemistry for TBEV antigen with a polyclonal Western-TBEV antibody (Dilution: 1:3000; source: Department of Virology; Medical University of Vienna) was performed with an automated Immunostainer (Thermo Autostainer 360-2D; Thermo-Fisher Scientific, Fremont; CA) using the UltraVision LP detection system (Thermo-Fisher) and DAB Plus as chromogen (Thermo-Fisher). As positive control brain of a TBEV-infected dog was used.

For parasitological analysis of the faeces, a flotation method with a zinc-chloride solution (specific gravity of 1.35) was used.

For detection of TBEV-specific RNA, two independent RT-qPCR protocols according to Schwaiger and Cassinotti [22], and adapted by Klaus et al. [23, 24], were applied. The TBEV strain Leila BH 95–15 was isolated from the brain material using BHK21 cells (Collection of Cell Lines in Veterinary Medicine (CCLV) 179, Friedrich-Loeffler-Institute, Greifswald-Insel Riems, Germany). The RNA of the brain sample was used for direct sequencing of the TBEV strain Leila BH 95–15. The complete coding sequence was generated by the use of primer-based Sanger sequencing (primer sequences available upon request). For phylogenetic analysis multiple alignments of 23 selected TBEV strains, Langat virus and the TBEV sequence from sheep Leila_BH95–15 were performed using the MAFFT method [25]. The evolutionary history was inferred using the neighbour-joining method [26]. The optimal tree with the sum of branch length = 1.19366931 is shown. The percentage of replicate trees in which the associated taxa clustered together in the bootstrap test (500 replicates) is shown next to the branches [27]. The tree is drawn to scale, with branch lengths in the same units as those of the evolutionary distances used to infer the phylogenetic tree. The evolutionary distances were computed using the Kimura 2-parameter method [28] and are in the units of the numbers of base substitutions per site. The rate variation among sites was modelled with a gamma distribution (shape parameter = 1). The analysis involved 25 nucleotide sequences. All positions containing gaps and missing data were eliminated. There were a total of 10,242 positions in the final dataset. Evolutionary analyses were conducted in MEGA6 [29]. Blood samples from the whole flock of sheep (*n* = 21) were collected and tested for TBEV antibodies by ELISA and serum neutralization test (SNT). A commercially available ELISA (Immunozym® FSME IgG all species, Progen Biotechnik GmbH, Heidelberg, Germany) was used, and sera were analysed according to the manufacturer’s protocol. Data were expressed as Vienna units (VIEU/ml). Values between 63 and 126 VIEU/ml were interpreted as borderline; lower and higher values were negative and positive, respectively.

In order to avoid false positive results of TBEV antibody titres detected in ELISA, all positive ELISA results of field collected samples were confirmed by the SNT as a gold standard according to a modified version of that established by Holzmann et al. [30]. For the SNT, the low pathogenic strain Langat was used with 100 TCID_50_/well. The virus titre used was confirmed by re-titrations. Serum samples were titrated in triplicates starting at a dilution of 1:5 in Minimum Essential Medium (MEM) Earle’s medium, and after a 24-h incubation time (37 °C), BHK-21 cell suspension was added to the virus-serum-sample and incubated at 37 °C for an additional 4 days. Subsequently, virus replication was detected by immunofluorescence analysis using a TBEV specific rabbit-antiserum. Titres were expressed as the reciprocal of dilutions that caused 50% neutralisation (ND50).

**Fig. 1:**
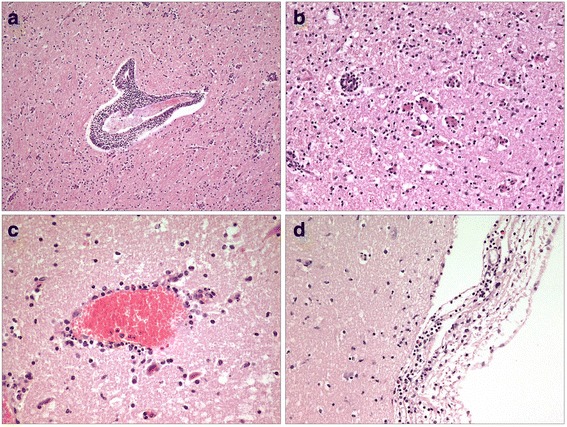
**a** Brain, thalamus, severe lymphohistiocytic perivascular infiltration of neuroparenchyma and gliosis, HE, 100 x. **b** Brain, thalamus, necrosis of neurons surrounded by neuronophagic nodules, HE, 200 x. **c** Brain, cerebral cortex, moderate lymphohistiocytic and neutrophil granulocytic perivascular infiltration of neuroparenchyma, HE, 400 x. **d** Leptomeninx, moderate, diffuse, lymphohistiocytic and neutrophil granulocytic infiltration, HE, 200 x

**Fig. 2:**
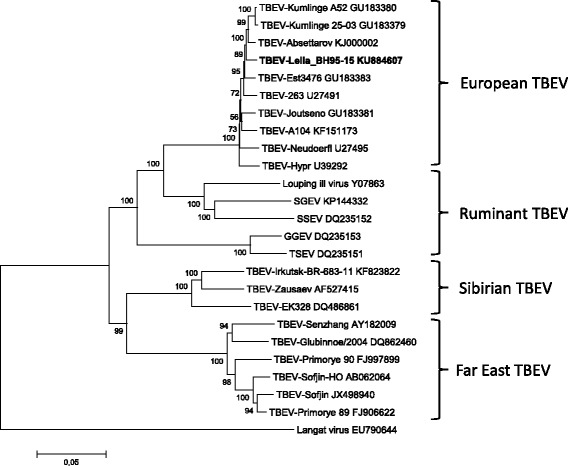
Phylogenetic analysis was performed with whole genome sequences and showed that TBEV Leila_BH95–15 belongs to the Eurasian TBEV clade. The optimal tree with the sum of branch length = 1.19366931 is shown

At necropsy and accompanying parasitology, the sheep showed a moderate to severe infestation with Trichostrongylids, Moniezia and Eimeria species. Histopathology of the brain revealed a mild to moderate, necrotising, lymphohistiocytic and granulocytic meningoencephalitis. The most severe inflammatory changes were noticed in the thalamus, hypothalamus and mesencephalon, followed by less severe lesions in the cerebral cortex, cerebral white matter and hippocampus as well as in the medulla oblongata, pons, cerebellum and leptomeninges. Frequent features were degeneration and necrosis of neurons surrounded by neuronophagic nodules as well as multifocal foci of gliosis. Inflammatory perivascular cuffs in the neuroparenchyma and diffuse inflammatory infiltrates in the leptomeninges were composed of mononuclear, lymphohistiocytic cells and a few neutrophils (Figs. 1a–d). Cytoplasmic or intranuclear inclusion bodies were not found. The cultured swab of the brain, especially for *L. monocytogenes*, was negative. Immunohistochemistry results for TBEV antigen detection were negative. The positive control (brain of a TBEV-infected dog) showed the expected specific staining. By TBEV quantitative RT-PCR, viral RNA was detected in brain samples of different locations and ascertained Cq values of 21 to 25. PCR analysis for Borna disease virus, West Nile virus and Louping ill virus scored negative. The brain sample was used for nearly complete sequencing of the TBEV genome (GenBank accession number KU884607). BlastN search revealed the highest nucleotide similarities of 98.63% for TBEV strain Kumlinge A52 (10,897 of 11,048 nt identical). The phylogenetic analysis confirmed the closest relation of the TBEV genome of the lamb to the classical European TBEV strains (Fig. 2). Virus isolation using BHK21 cells finally resulted, in the third passage, in a high-titre stock (10^7^ TCID_50_ ml^−1^) of the sheep isolate Leila BH95–15. Serum samples from individual sheep of the herd were tested for TBEV specific antibodies. One sheep serum of the flock tested positive for TBEV antibodies, and one tested as borderline for TBEV antibodies. The positive one of these two sera was confirmed as specific TBEV positive in SNT (ND50 1:30).

TBEV infection was diagnosed based on the clinical symptoms, the histopathology of the brain and the detection of TBEV in the brain via RT-qPCR and subsequent sequencing.

## Discussion and conclusions

To the authors’ knowledge, this is the first report of a natural TBEV infection in a sheep in Europe with clinical manifestation. Single cases of TBEV infection have been described in dogs [8–10], horses [11–13], small ruminants [20, 21, 31, 32] and a monkey [14, 15]. The number of unreported/undetected cases remains unclear as the clinical signs, albeit neurological, are non-specific and maybe interpreted by owners and veterinarians most likely as a *L.monocytogenes* infection. Since several animals of the flock had ticks, it can be assumed that the sheep was infected at the pasture by a tick bite. The species in this specific case was not determined, but large domestic animals, such as goats, sheep and cattle, are potential hosts for *I. ricinus*. Possible TBEV prevalence in this area was also confirmed by the TBEV antibody titre of one further sheep in the flock. Interestingly, during experimental inoculation using TBEV sheep and goats showed almost no clinical symptoms except fever [33, 34]. Only a minority of TBEV-positive small ruminants displayed prominent neurological signs [20, 31, 32]. Additionally, Bagó et al. [21] reported only a moribund condition of the TBEV-positive mouflon. Further investigations are necessary to evaluate the relevant factors, such as host immunity, co-infections (e.g. parasitic infestation), age, TBEV strain and virus load, causing clinical signs in small ruminants.

Neuropathological lesions of this case resemble these in human beings [35] and in wild and domestic animals [10, 11, 14, 21] after TBEV infection. A glial shrubbery as described by Bagó et al. [21] and Weissenböck et al. [10] was not observed. The presence of a pronounced neuronophagia and numerous resulting glial nodules corresponds well with a protracted disease course. This may explain the negative outcome of immunohistochemical antigen detection because rapid virus clearance is a typical feature of TBE [10]. Borna disease-, rabies-, scrapie-, listeriosis associated histopathological lesions were not found. Other potential flavivirus infections with similar histopathological lesions for example Louping ill disease (dramatic clinical course, up to now not yet reported in Germany) and West Nile Encephalitis (not yet reported in Germany) were ruled out by PCR.

Sheep or goat flocks, especially those that are used for the production of raw milk or raw milk products and live on pastures with possible tick contact, should be tested for TBEV, because these can be the source of transmission of so-called alimentary TBE to humans. Experimental investigations showed that during the viraemic stage, the virus is excreted in milk for 3–7 days and consumption of non-pasteurised milk or milk products might lead to infection [16, 33, 36, 37]. In the past few years, alimentary TBE cases in humans were reported from Slovakia, Poland, Estonia, Austria and Hungary [2, 3, 38–41]. In Germany the first case of an alimentary TBEV infection was reported in June 2016 [42]. Two persons in Reutlingen, a TBE risk area of Baden-Wuerttemberg, which is 68 km away from the location of the reported case, were affected by consumption of raw goat milk products. However, individual cases or small group outbreaks in humans may be possible. Therefore, consumption of raw milk should be avoided to reduce the risk of human infection. In Hungary, after an outbreak of TBEV in a milking goat, flock animals were successfully immunised against TBEV to allow further raw milk production connected with grazing on pastures [34]. Besides the very rare clinical symptoms in sheep and goats, serological investigations can provide information about possible infection with TBEV in an area. It is an inexpensive method to improve consumer’s protection against TBE in those cases when milking goats and sheep (very seldom cows), especially in TBE risk areas, but also in areas with only single human TBE cases [43]. As the viraemic period is very short and clinical symptoms might be absent or misinterpreted, testing of TBEV specific antibodies could highlight the TBEV specific epidemiological situation of a flock.

For neurological symptoms in sheep, especially in TBE risk areas, TBEV infection should be taken into consideration, especially in cases where other diseases with neurological symptoms were excluded, for example Louping ill disease, Borna disease, rabies, scrapie, listeriosis, tetanus, malnutrition and intoxications.

## Abbreviations

DAB: Diaminobenzidin
ELISA: Enzyme-linked Immunosorbent assay
HE: Hematoxylin and eosin
I.: Ixodes
L.: Listeria
RNA: Ribonucleic acid
RT-qPCR: Quantitative reverse transcriptase Polymerase chain reaction
SNT: Serum neutralisation test
TBE: Tick-borne encephalitis
TBEV: Tick-borne encephalitis virus
VIEU: Vienna units
